# Families’ Degree of Satisfaction With Pediatric Telehomecare: Interventional Prospective Pilot Study in Catalonia

**DOI:** 10.2196/17517

**Published:** 2020-03-26

**Authors:** Francesc López Seguí, Astrid Batlle Boada, Juan José García García, Ana López Ulldemolins, Ane Achotegui del Arco, Cristina Adroher Mas, Francesc García Cuyàs

**Affiliations:** 1 TIC Salut Social Ministry of Health Barcelona Spain; 2 Centre for Research in Health and Economics Department of Experimental and Health Sciences Pompeu Fabra University Barcelona Spain; 3 Sant Joan de Déu Hospital Catalan Ministry of Health Barcelona Spain

**Keywords:** home health monitoring, pediatrics, telehomecare, videoconferencing, satisfaction with care, remote sensing technology, telemedicine, telehealth

## Abstract

**Background:**

Pediatric home hospitalization improves the quality of life of children and their families, involving them in their children’s care, while favoring the work-life balance of the family. In this context, technology guarantees accessibility to assistance, which provides security to users. From the perspective of the health care system, this could lower the demand for hospital services and reduce hospitalization costs.

**Objective:**

This study aimed to assess families’ degree of satisfaction and acceptability of pediatric telehomecare and explore the clinical characteristics of children benefiting from the program.

**Methods:**

A total of 95 children and their families participated in the home-hospitalization pilot program operated by Sant Joan de Déu Hospital in Barcelona, Spain. Families were visited once a day and patients were monitored using a kit consisting of a scale, a thermometer, a pulse oximeter, and a blood pressure monitor. Data on parental experience, satisfaction, safety, and preference for care was collected by means of a questionnaire. Data about the children’s characteristics were collected from medical records. Descriptive and comparative statistics were used to analyze the data.

**Results:**

A total of 65 survey respondents expressed very high levels of satisfaction. Families reported their experiences as being very positive, preferring home hospitalization in 94% (61/65) of cases, and gave high scores regarding the use of telemonitoring devices. The program did not record any readmissions after 72 hours and reported a very low number of adverse incidents. The user profile was very heterogeneous, highlighting a large number of respiratory patients and patients with infections that required endovenous antibiotic therapy.

**Conclusions:**

Pediatric home hospitalization through telemonitoring is a feasible and desirable alternative to traditional hospitalization, both from the perspective of families and the hospital. The results of this analysis showed a very high degree of satisfaction with the care received and that the home-based telemonitoring system resulted in few adverse incidents.

## Introduction

Pediatric home-based care is a good alternative to conventional hospitalization insofar as it is consistent with a care model that places a high value on a more humanized form of health care and encourages self-care and children’s rights. The provision of this type of care for children with acute and chronic illnesses is increasing in western countries due to technological developments [1], improvements in support services [2], rising health care costs [3], and the potential psychosocial benefit for children and their families [4].

The technology available today allows for remote and real-time monitoring of a patient’s clinical status and regular follow-up with families. Developments in health care equipment means that many diagnostic and treatment procedures normally conducted in a clinical environment can be provided at home [5,6]. Likewise, the increase in the survival of severe processes (ie, complicated interventions that have caused death in the past) and the greater availability of treatments for patients affected by rare diseases has increased the cohort of fragile patients and/or those who are in need of follow-up care; in all likelihood, the hospital environment is not the best place to look after them. This means there is a contingent of stable patients, who are not outpatients, who need prolonged hospital stays in order to complete treatments. Home-based hospitalization care can prevent hospital admission or shorten the average stay.

Home is a child’s natural environment. The European Association for Children in Hospital Charter establishes that a child should only be admitted to hospital if it is absolutely necessary and must be discharged as soon as possible [7]. Earlier studies show a high degree of satisfaction among pediatric patients and their families when hospitalized at home [8-14]. Additionally, hospital facilities, especially those located in urban and highly complex environments, see the need to rationalize their spaces. Freeing up some hospital beds by sending patients home could be a good response to the growing demand and the increase in the complexity of the cases dealt with [15,16].

In this context, the Sant Joan de Déu Hospital in Barcelona decided to initiate a pilot program on pediatric home-based hospitalization care. This study aims to (1) measure the impact of the intervention on the satisfaction of patients and their families and (2) determine the clinical and sociodemographic characteristics of the children benefiting from the program in view of the possible deployment of the intervention.

## Methods

### Setting

The Sant Joan de Déu Hospital in Barcelona is a third-level university hospital located in Catalonia, Spain, which specializes in the fields of pediatrics, gynecology, and obstetrics. It is a privately owned hospital that operates as part of the public health system and the Catalan hospital network. It sees approximately 27,000 cases annually, with around 250,000 outpatient consultations; 15,000 surgical interventions; and 160,000 emergencies. The study involving the pediatric home-based care pilot program took place between April 1 and June 30, 2019. The candidate users were selected in accordance with the criteria outlined in Textbox 1.

**Selection criteria for pediatric home hospitalization.**

- Distance: patient’s home is no more than 30 minutes from the hospital
- Clinical stability: patient is stable without forecasting decompensations in the short term
- Voluntary consent is given by the family and, where applicable, by the child
- Habitability conditions of the home: composition of the family group, individual room for the patient, cleanliness condition of the home, availability of the minimum infrastructure for the patient’s personal hygiene, and the ability to comply with the prescribed diet, environmental conditions of noise, and ambient temperature
- Prior family training to ensure continuity in the care process
- Possibility of establishing permanent telephone communication

When the medical team detected a potential case, they contacted the home-hospitalization team, who assessed it and made sure it met the selection criteria. In that case, the family was informed of what home hospitalization involves and was provided with information in writing. If the family agreed, they were asked to give informed consent. Finally, the team’s nurse trained the family and empowered them to carry out the necessary care; when leaving, they were issued a kit (see Table 1) containing devices for remote telemonitoring—thermometer, pulse oximeter, blood pressure monitor, and scale—together with a tablet, which used Bluetooth and special software to record information registered by the devices and enabled videocalls.

**Table 1:** Contents of the remote telemonitoring kit.

Device	Brand (manufacturer)	Model	Medical device certification
Blood pressure monitor	iHealth View (iHealth Labs)	BP75	Yes
Pulse oximeter	iHealth Air (iHealth Labs)	P03M	Yes
Scale	iHealth Lina (iHealth Labs)	HS2	No
Thermometer	OMRON (Omron Healthcare)	GentleTemp 521	Yes
Tablet	iPad (Apple)	MR6P2TY/A	No

The intervention considered two types of complementary health care: face-to-face, with a daily visit, and telecare (ie, 24/7 continuous care via remote real-time monitoring, phone calls, and videoconferencing). The human resources devoted to the project were 1 pediatrician, 2.7 nurses, technical support, and part-time administrative staff. Clinicians traveled from the hospital by means of a car and there were 10 remote telemonitoring kits.

### Data Collection and Outcome Measures

Once the intervention was finished, an ad hoc, nonvalidated, and self-administered survey was conducted using Google’s online survey tool (see Multimedia Appendix 1); the survey included multiple variables related to satisfaction and participants used the tablet provided to complete the survey. This questionnaire did not include names, medical record numbers, or any data that could identify the participants. The analysis of the results did not require any kind of user identification. The following user clinical and sociodemographic information data were extracted from the administrative database and transferred to a designated form: age, sex, source of referral, medical specialty, main caregiver, child location, type of intravenous line, administration schedule, readmission date, and reason for readmission.

This was a unicentric, single-arm, interventional prospective study with no control group. The statistical program R, version 3.6.1 (The R Foundation), was used for the statistical analyses.

### Ethical Considerations

The study was approved by Sant Joan de Déu Hospital’s Ethical Committee for Clinical Research (registration No. 88-19) and was carried out in accordance with the Helsinki Declaration [17].

## Results

### Characteristics of the Beneficiaries

Participant characteristics are shown in Table 2. The typical profile of a home-based hospitalized patient in our study was a 4-year-old boy (53/95, 56%) who lived 12 km from the hospital, was previously hospitalized (85/95, 89%) in the pediatrics department (80/95, 84%), and whose main caregiver was their mother (54/95, 57%). They returned home with an intravenous inserted (48/95, 51%) and their administration schedule was every 24 hours (36/89, 40%). A total of 89% (85/95) of patients included in this study came from hospitalization, 8% (8/95) came from outpatient visits, and 2% (2/95) came from the emergency department. The clinical profile was diverse, with the most frequent pathologies being infectious diseases that required endovenous antibiotic therapy, head and neck infections (ie, adenitis, adenophlegmons, and mastoiditis), pneumonia, urinary tract infections, and respiratory infections requiring bronchodilator nebulization and/or oxygen therapy (1 L/min or less, administered via nasal cannula). To a lesser extent, fever without a focus was treated in infants under observation, atypical febrile convulsion was treated under observation, and endovenous serotherapy was given in cases of dehydration.

The cohort studies did not show any security incidents related to medication administration. However, there were 4 readmitted patients out of 95 (4%). In 2 cases, readmission was due to the evolution of the disease (ie, a nephrotic syndrome that developed into a bronchospasm and a peritonsillar phlegmon due to poor control of pain). After these 2 readmissions, some adjustments were made to minimize problems that could have been prevented; this included a deeper interview with families, explaining how the program works and what the terms and conditions are. Also, patients who required oxygen were not discharged from hospital until the oxygen supply was at home.

In 1 case, a bronchospasm occurred because a supply of oxygen was not provided during the home hospitalization. In this case, the bronchospasm occurred due to a nephrotic syndrome caused by the lack of compliance with the medical indications at home. The patients who were hospitalized with the peritonsillar phlegmon due to poor control of pain, and the bronchospasm due to lack of oxygen, returned home the next day. These readmissions should be interpreted as a sign of program success, because each family freely decided to resume home hospitalization. In the cases of poor control of pain and the lack of oxygen supply, once controlled, the family felt secure to go home.

### Satisfaction Results

Survey results regarding general satisfaction with the intervention are reported in Table 3. Of the 95 patients included in the program, 65 completed the satisfaction survey (68%); of these, only 3% (2/65) indicated they had more work than what they had expected, only 3% (2/65) would have preferred conventional hospitalization, and 100% (63/63) would repeat the experience. Level of care was scored overall as *Excellent* (60/65, 92%); the information provided by the staff during home hospitalization was also scored as *Excellent* (54/65, 83%).

Most of the respondents (49/64, 77%) received their first home visit less than 24 hours following their home hospitalization and did not have to call to ask for help (35/65, 54%); for those who did ask for help, the problem was resolved quickly (30/33, 91%). They valued the fact that the pediatrician and the nurses worked in a coordinated way and that their home visit lasted a sufficient amount of time (65/65, 100%).

**Table 2:** Sociodemographic characteristics of the sample.

Characteristic		Values (N=95)	
**Gender, n (%)**			
	Total		95 (100)
	Boy		53 (56)
	Girl		42 (44)
Age (years), mean (SD)		4.22 (4.57)	
**Source of referral, n (%)**			
	Total		95 (100)
	Hospitalization		85 (89)
	Outpatient visits		8 (8)
	Emergencies		2 (2)
**Medical field, n (%)**			
	Total		95 (100)
	Pediatrics		80 (84)
	Nephrology		6 (6)
	Surgery		5 (5)
	Orthopedic surgery and traumatology		2 (2)
	Gastroenterology		1 (1)
	Others		1 (1)
Distance (km) to hospital, mean (range)		11.72 (10-50)	
**Main caregiver, n (%)**			
	Total		95 (100)
	Mother		54 (57)
	Mother and father		35 (37)
	Father		5 (5)
	Other		1 (1)
**Type of intravenous line, n (%)**			
	Total		48 (100)
	Peripheral route		44 (92)
	Peripherally inserted central catheter		2 (4)
	Broviac		1 (2)
	Midline		1 (2)
**Schedule of administration, n (%)**			
	Total		89 (100)
	Every 24 hours		36 (40)
	Every 8 hours		22 (25)
	Every 4 hours		21 (24)
	Every 6 hours		7 (8)
	Every 12 hours		2 (2)
	Continuous		1 (1)

**Table 3:** Survey results regarding general satisfaction with the intervention.

Survey question and responses		Participants (N=65), n (%)
**How many days passed since your child was discharged from hospital until their first visit home?**		
	Total	64 (100)
	Between 1 and 3 days	15 (23)
	Less than 1 day	49 (77)
**Did you have to call to ask for help in relation to any problem with your child while he or she was hospitalized at home?**		
	Total	65 (100)
	Yes	30 (46)
	No	35 (54)
**If so, was the problem resolved quickly?**		
	Total	33 (100)
	Yes	30 (91)
	No	3 (9)
**Do you think that the pediatrician and the nurses worked in a coordinated way?**		
	Total	65 (100)
	Yes	65 (100)
	No	0 (0)
**Do you think that the staff spent enough time with your child and family during their home visits?**		
	Total	65 (100)
	Yes	65 (100)
	No	0 (0)
**How would you rate the way in which the staff has taken care of your child and family?**		
	Total	65 (100)
	Excellent	60 (92)
	Very good	5 (8)
**How would you rate the information provided to you by the home-hospitalization team during the home-based care?**		
	Total	65 (100)
	Excellent	54 (83)
	Very good	11 (17)
**In relation to your child’s home-based care and regarding the duties that you normally assume, what has the amount of work been like?**		
	Total	65 (100)
	As expected	49 (75)
	Less than expected	14 (22)
	More than expected	2 (3)
**Would you have preferred conventional hospitalization instead of your child being at home?**		
	Total	65 (100)
	Yes	2 (3)
	No	61 (94)
	I don’t know	2 (3)
**If necessary, would you like your child to be taken care of by the home-based care team again?**		
	Total	63 (100)
	Yes	63 (100)
	No	0 (0)

Survey results regarding satisfaction with the devices are reported in Table 4. Regarding the use of the telemonitoring devices—thermometer, pulse oximeter, blood pressure monitor, scale, and tablet—results show that the software was perceived as *Easy* (47/58, 81%) and 91% of respondents (49/54) were able to take the corresponding vitals easily. The scores, measured from 0 (*Very bad*) to 5 (*Excellent*), regarding communication with the clinical team and regarding the devices—scale, thermometer, pulse oximeter, and blood pressure monitor—were very high (range 3.79-4.61). In a qualitative assessment space, it was mentioned that the scale was the least useful device and respondents experienced problems with the thermometer because it was not fully adapted to the physiology of the pediatric users.

**Table 4:** Survey results regarding satisfaction with the devices.

Survey question and responses		Values (N=65)
**How would you rate the software’s accessibility? n (%)**		
	Total	58 (100)
	Easy	47 (81)
	Neither easy nor difficult	3 (5)
	I did not access the program	8 (14)
**Have you been able to easily take the vitals that you have been asked to take? n (%)**		
	Total	54 (100)
	Yes	49 (91)
	No	5 (9)
How would you rate the communication with the clinical team using this tool? (n=59), mean score^a^ (SD)		4.61 (0.65)
How would you rate the utility of the videoconferencing sessions? (n=57), mean score (SD)		4.28 (1.02)
Of the devices you were issued, together with the tablet, how would you rate the scale? (n=38), mean score (SD)		4.25 (1.02)
Of the devices you were issued, together with the tablet, how would you rate the thermometer? (n=48), mean score (SD)		3.79 (1.36)
Of the devices you were issued, together with the tablet, how would you rate the pulse oximeter? (n=45), mean score (SD)		4.21 (1.12)
Of the devices you were issued, together with the tablet, how would you rate the blood pressure monitor? (n=45), mean score (SD)		4.19 (1.17)

^a^Scores were measured on a scale from 0 (*Very bad*) to 5 (*Excellent*).

The results of this analysis showed a high degree of satisfaction with the care received and highlight the fact that the telehomecare system did not generate significant adverse incidents. Overall, the intervention (ie, training, face-to-face visits, and telemonitoring) enabled the families to be self-sufficient regarding their children’s care. Their satisfaction with the devices was very good and their perception of accessibility was regarded as excellent.

## Discussion

### Principal Findings

This study assessed the impact of the pediatric, home-hospitalization, pilot program of the Sant Joan de Déu Hospital in Barcelona on the satisfaction of patients and their families; the study also assessed the clinical and sociodemographic characteristics of the children benefiting from the program in view of the potential deployment of the intervention. Although a small sample has been studied, the experience suggests that the intervention could be extended to patients originating from specialties other than the pediatric specialty (ie, surgery, orthopedic surgery and traumatology, gastroenterology, and nephrology).

### Limitations

During the pilot study, a problem with the size of the devices was identified, as they are not always suited to the physiology of pediatric patients, meaning the families used them less. This factor should be taken into account in view of the possible extension of the intervention in the hospital itself or in any replication of the experience.

Finally, the guarantee of the anonymity of the information gathered by the survey has made it impossible to cross-reference this data with administrative data. Future studies should examine the differential impacts on satisfaction according to type of illness or other sociodemographic factors.

### Conclusions

Pediatric home-based care is preferred by patients and their families. Remaining in their homes and staying in their environments contributes to patient-centered care, while empowering the patients and their families in the care and control of their illnesses. In keeping with the evidence already published, this study shows that home-based hospitalization is associated with an improvement in the quality of life of the child and the family and with a potential decrease in the demand for hospital services. Telemonitoring tools are one of the essential elements that make this possible. The high degree of acceptance of the devices—thermometer, pulse oximeter, blood pressure monitor, and scale—is an opportunity to study the implementation of new tools that reinforce and offer guarantees of certain types of care.

In terms of the impact on clinical outcomes, future studies should determine whether, as with the adult population, clinical outcomes are comparable to or better than those of conventional hospitalization by analyzing the impact on readmission or mortality with respect to the usual path of hospitalization. Likewise, we must study the cost-effectiveness of this type of intervention, by comparing the cost of travel and that of the devices with the savings derived from the reduction of days in hospital, reduction of conventional hospitalization costs, and the increase in hospital capacity resulting from the freeing up of beds.

## Appendix

Multimedia Appendix 1 Self-administered study survey.
