# Supplementary material for: Families’ Degree of Satisfaction With Pediatric Telehomecare: Interventional Prospective Pilot Study in Catalonia
Source: JMIR Pediatr Parent. 2020 Mar 26;3(1):e17517. doi: 10.2196/17517 (PMC7146252; doi:10.2196/17517)
Supplement: Multimedia Appendix 1 [file pediatrics_v3i1e17517_app1.docx]

A

| **Appendix: Survey** |
| --- |

SJD A CASA: Analysis of the home-based-care hospitalization and the degree of satisfaction of patients and their families.

Below you will find an anonymous survey that we would appreciate that you answered to. The objectives of the survey are to assess the healthcare processes and the degree of satisfaction that your child and family have had during the home hospitalization. The information which you provide in this survey will be completely confidential. Thank you very much in advance.

We will now ask you some general questions about the information that was provided to you before the home hospitalization:

[1] Did the *SJD a Casa* team (the pediatrician and nurses who followed-up your child home) clearly explain what the home-based care program was like?

Yes

No

I don’t know

[2] Did you sign an informed consent document?

Yes

No

I don’t know

[3] Did the *SJD a Casa* team explain to you how to take care of the child at home, any possible complications and how to act if they were to occur?

Yes

No

I don’t know

[4] Did they give you the 24 hours contact telephone number?

Yes

No

I don’t know

Now we will ask you some questions about the coordination of the *SJD a Casa* team:

[5] Has the nursing team visited you home?

Yes

No

I don’t know

[6] Has the pediatrician visited you home?

Yes

No

I don’t know

[7] How many days passed since your child was discharged from hospital and the first visit home?

Less than 1 day

Between 1 and 3 days

More than 3 days

[8] Have you had to call the telephone number given to you due to problems related to your child’s home-based-care hospitalization?

Yes

No

I don’t know

[9] If so, was the incident or problem resolved?

Yes, quickly

Yes, but it took a while

It was not resolved

[10] Do you think that the pediatrician and the nurses worked in a coordinated way?

Yes

No

I don’t know

[11] Are you satisfied with the number of visits made by the nursing team?

Yes

No

I don’t know

[12] Are you satisfied with the number of visits made by the pediatrician?

Yes

No

I don’t know

[13] Do you think that the time spent by the *SJD a Casa* staff during their home visit lasted sufficient?

Yes

No

I don’t know

[14] Did the home-based-care staff tell you their names?

Yes

No

I don’t know

[15] How would you rate the way in which the *SJD a Casa* staff has treated your child and your family?

Excellent

Very good

Good

Not so good

Very bad

I had no contact

I don’t know

[16] How would you rate the information provided to you by the *SJD a Casa* staff during the home-based care?

Excellent

Very good

Good

Not so good

Very bad

I had no contact

I don’t know

[17] On the last day of the home-based care, were you informed of the discharge?

Yes

No

I don’t know

[18] On the last day of the home-based care, were you provided with a discharge report?

Yes

No

I don’t know

[19] Regarding the work at home that you have had to do, and in relation to the duties you take care of, what has the amount of work been like?

More than expected

As expected

Less than expected

[20] If to the previous question you answered “More than expected”, what is the reason of it, according to you?

[21] Would have you preferred your child to be admitted in hospital instead of being admitted home?

Yes

No

I don’t know

[22] If you answered yes, indicate the reason for it:

For peace of mind and safeness.

Because in hospital the child would have been better looked after.

Because the child was not well enough to keep up an entry home.

Others

[23] If to the previous question you answered “Others”, please tell us what do you refer to:

[24] Overall, how do you rate the care given by *SJD a Casa*?

Excellent

Very good

Good

Not so good

Very bad

I had no contact

I don’t know

[25] If it were necessary, would you like your child to be taken care of by the *SJD a Casa* team again?

Yes

No

I don’t know

[26] Do you think there is something which could be improved regarding the care given during the home hospitalization?

[27] How did you feel during the home hospitalization?

[28] How did your child feel during the home hospitalization?

[29] If you have any other comments, you can write them here:

[30] Have you used the REVITA program (on the tablet) during the home hospitalization?

Yes

No

I don’t know

[31] How would you rate access to the REVITA program?

Easy

Neither easy nor difficult

Difficult

I haven’t been able to access it

[32] Have you been able to take the vitals that you have been asked for easily?

Yes

No

I don’t know

[33] How would you rate communication with the *SJD a Casa* healthcare team through this tool?

Excellent

Very good

Good

Not so good

Very bad

I had no contact

I don’t know

[34] How would you rate the utility of the videoconferencing?

Excellent

Very good

Good

Not so good

Very bad

I had no contact

I don’t know

[35] Of the devices you were issued with together with the tablet, how would you rate them? (Scale, thermometer, pulse oximeter, blood pressure monitor)

Excellent

Very good

Good

Not so good

Very bad

Haven’t used it

I don’t know

Thank you very much!
